# Corrigendum to "Postpartum health of working mothers: A prospective study"

**DOI:** 10.51866/cor.001

**Published:** 2023-11-24

**Authors:** Janting Majorie Ensayan Anak, Whye Lian Cheah, Hazmi Helmy

**Affiliations:** 1 PhD (USM), Department of Community Medicine and Public Health, Faculty of Medicine and Health Sciences, Universiti Malaysia Sarawak, Kota Samarahan, Sarawak, Malaysia. Email: wlcheah@unimas.my; 2 DrPh (UNIMAS), Department of Community Medicine and Public Health, Faculty of Medicine and Health Sciences, Universiti Malaysia Sarawak, Kota Samarahan, Sarawak, Malaysia.; 3 MCommMed (USM), Department of Community Medicine and Public Health, Faculty of Medicine and Health Sciences, Universiti Malaysia Sarawak, Kota Samarahan, Sarawak, Malaysia.

In our article titled "Postpartum health of working mothers: A prospective study," publishedin this journal (Malays Fam Physician (2023;18:48)),^1^ we would like to bring to the readers' attention to certain discrepancies that have come to light.

The Editorial Board of Malaysian Family Physician received feedback highlighting similarities between our aforementioned article and another publication titled "Depression level and its associated factors among postpartum working women in Kuching, Sarawak - a cross-sectional study," published in Malays J Med Sci (2023;30(4):147-56).^2^

We would like to clarify that the two papers indeed share data, but they serve different research objectives. The first article utilized a prospective study design, aiming to investigate postpartum depression over a span of 6 and 12 weeks postpartum. In contrast, the second article employed a cross-sectional study design, focusing solely on the first 6 weeks postpartum to explore factors contributing to maternal depression levels. We have cited the cross-sectional study and included the following sentence in the Methods section of the prospective study published in this journal: "Parts of the study data have been reported in a previous publication".

We acknowledge the oversight in not explicitly addressing the data overlap in the respective publications. We assure our readers that the objectives, study designs, and data analyses of both articles remain distinct.
